# Effect of Consumption Value on Consumer Willingness to Consume GM Food: A Post-COVID-19 Analysis

**DOI:** 10.3390/foods11182918

**Published:** 2022-09-19

**Authors:** Muhammad Ghufran, Jawaria Ashraf, Sumran Ali, Peng Xiaobao, Luigi Aldieri

**Affiliations:** 1 Department of Economic and Statistical Sciences, University of Salerno, 84084 Fisciano, Italy; 2 School of Public Affairs, University of Science and Technology of China (USTC), Hefei 230026, China; 3 School of Management, University of Science and Technology of China (USTC), Hefei 230026, China; 4 School of Intellectual Property Rights, University of Science and Technology of China (USTC), Hefei 230026, China

**Keywords:** consumption values, food attitude, consumer animosity, consumer ethnocentrism, USA, China

## Abstract

In this research, we debate the critical challenges posed by the COVID-19 pandemic such as food scarcity, by examining the influence of consumption values on consumers’ willingness to consume genetically modified (GM) food in the presence of consumer food attitudes, animosity, and ethnocentrism, which could be the one possible option to deal with the food scarcity problem. The proposed relationship could help to understand the complex societal problem of food scarcity and import dependency in the food sector before and after the crisis. Therefore, based on the theory of consumption values, we investigated government actions, consumer attitudes, and their willingness to consume GM food through 1340 valid USA responses and 1065 Chinese responses. We observed that COVID-19 doubled the number of malnourished people in 2020 relative to 2019, while consumption values, to some extent, changed consumer food attitudes and were inclined toward other food alternatives such as GM food regardless of governmental support for GM food in both USA and China. Moreover, this research enables governments, policymakers, market practitioners, and other stakeholders to use the COVID-19 crisis as an opportunity to negotiate with other countries to share their food technology along with imports.

## 1. Introduction

COVID-19 has had a devasting impact on the economic and health sectors, including food security, safety, and hygiene; thus, exposing our so-called highly organized and modernized system. However, the COVID-19 crisis substantially changed consumer perceptions and attitudes toward organic food, which is considered to be safe and healthy but costly and not easily accessible according to Ghufran, et al. [1], especially in China. In addition, the world faced a food shortage problem even before the COVID-19 crisis; as per the United Nations report of 2019, 690 million people were malnourished, which was the 8.9% of the entire world population [2], and the COVID-19 outbreak doubled that figure, placing an extra 130 million people at risk of severe starvation by the end of 2020 [3]. As predicted by the World Food Program (UN), the world’s population has been estimated to reach 9 billion by 2050, which means that GM food might be a potential solution to alleviate hunger in developing and underdeveloped nations [3,4].

Therefore, genetically modified (GM) food has proliferated in developed and developing countries since its introduction in 1992. There was a 10-fold increase in the amount of land dedicated to GM food between 1996 and 2018. For example, GM food cultivation started by encompoassing 1.7 million hectares in 1996, covered 191.7 million hectares in 2018, and reached 2.7 billion hectares in 2019 which was a substantial increment in the outreach of the GM food sector [5] and helpful for food scarcity as per population growth. At the same time, the USA led the GM food market from 1996 to 2017 with the production of nine GM crops (soybeans, maize, cotton, alfalfa, canola, sugar beets, potato, apples, squash, and papaya) on 75 million hectares [6] and developed a substantial export to China [7]. Here, consumers have shown some skepticism about food and technology overindulgence because of this rapid expansion [8] and the perceived risks of GM foods to the environment and human health [9,10]. Therefore, the scientific community has begun debates regarding genetically modified organisms’ potential advantages and hazards (GMOs), leading to consumer apprehension about GM food and technology [11]. Recent survey data revealed widespread consumer concern over GM foods throughout the world, particularly in China and America, even though GM maize, soybeans, canola, GM papaya and rice were among the most often imported and consumed GM goods in China [12,13]. According to Chinese consumer research, only 11.9 percent of Chinese consumers have a good attitude toward GM food [14]. In contrast, the United States (USA) is the world’s leading manufacturer and producer of GM food, accounting for 73.1 million hectares of land and 40% of the global GM food output, whereas only 51 percent of its citizens believe GM food is safe [15]. However, both governments support the idea of GM food in their respective jurisdictions, but consumer reactions are different [16]. There is a significant gap in the economics and consumer responses to GM foods. The risks connected with GM foods are more significant than those associated with GM technology used in the medical realm [17]. Moreover, consumer worry over the role of technology in food production has led to the establishment of complicated and expensive food safety and labeling requirements [18]. The research has emphasized the critical relevance of understanding the elements that contribute to public acceptance of GM foods. In this study, we aim to demonstrate the effectiveness of a psychologically integrated model that extends the theory of consumption values (TCV) and food attitude in light of consumer readiness to consume GM foods in the world’s two most economically powerful countries.

Furthermore, the willingness to consume a product is identified as the most potent predictor of behavioral outcomes [19] influenced by the consumer’s attitudes [20] toward the food products, which could be consumer animosity and ethnocentrism [21]. Additionally, an attitude refers to a consumer’s psychological willingness to prefer or hate a GM food product. Similarly, consumer perceptions regarding food products are shaped by consuming values, for example, functional attributes such as price and nutritional determinants [1,22]; social acceptance and recognition of food products [23,24]; environmental concern and social standing [25]; and consumer knowledge [26]. These consuming values significantly influence consumers’ attitudes [27,28] that compel them to purchase GM food products.

This study’s primary contribution is to investigate the complex societal problem of food scarcity and import dependency in the food sector before and after the COVID-19 pandemic crisis by examining how consumption values influence consumer willingness to consume GM food, which could be a viable solution to deal with the food scarcity problem. For this reason, we demonstrate the effectiveness of a psychologically integrated model that extends the TCV along with its four consumer values with subdivisions as functional (price and nutrition), emotional (environmental), social (social approval), and epistemic (perceived GM food knowledge) to examine consumer consumption willingness to consume GM foods in the presence of consumer animosity and ethnocentrism in the world’s two most economically powerful countries. Second, this study enhances the knowledge and preferences of practitioners, policymakers, and academics by assisting them in identifying which values underlie certain consumption choices. Thirdly, we attempt to identify the mediation function of ethnocentrism and consumer animosity against consumer willingness to consume GM food.

## 2. Theoretical Framework and Hypotheses

**Figure 1:**
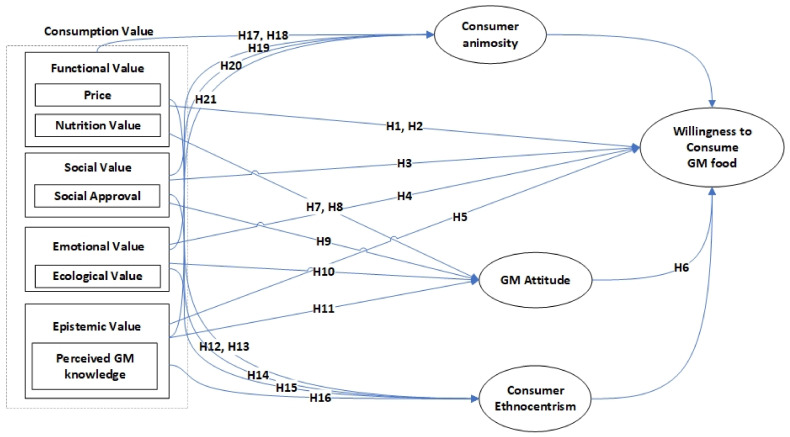
Research framework.

In this section, we examine the willingness to consume GM food using the TCV, consumer ethnocentrism, and animosity as evidenced in the literature to support the proposed relationship (see Figure 1). Figure 1 shows the conceptual framework for this research.

### 2.1. Consumption Values

The consumption value theory focuses on consumption values that conceptualize consumer behavioral responses towards products, such as why people purchase or do not purchase goods, why people prefer one kind of goods over another, or why people prefer a particular brand over another [29,30]. These consumption values consist of five distinct values [29]: functional, social, emotional, conditional, and epistemic. These values all have essential functions to play, and each value has a notable impact on a person’s behavior and attitude [31]. In this study, we considered these consumption values to investigate consumer preferences and willingness to purchase GM food in the presence of consumer ethnocentrism and animosity, during the COVID-19 crisis when consumers were more conscious about their health and food safety and seemed to be more inclined towards other food options that were considered to be safe and healthy [1].

In the following section, we introduce values and the willingness to consume GM foods as discussed in the literature.

### 2.2. Functional Value

The concept of functional value has previously been utilized in the food sector [32] as a product’s functional capabilities [33] in terms of price and quality [34]. It is also considered to be a fundamental value in consumer decisions to prefer or neglect food products [35]. According to the previous literature, consumers usually undertake price comparisons when the perceived prices of food products are greater than expected, and they prefer to purchase more nutritious and healthy food [1,36]. More specifically, the literature on functional values exhibits two sets of thought in the food sector: First, consumers are willing to pay premium rates for high-quality and nutritious food options [37], in fact, consumers are willing to spend 37% more for hygiene and nutritious food [38]. Secondly, consumers are unwilling to pay premium prices for scientifically proven nutritious GM food [39] because they have a trust deficit [40] and due to controversies on social media about GM food [41]. Therefore, it is a challenge for manufacturers and producers to maintain reasonable prices and quality of GM foods that are similar to conventional and organic foods. For instance, in Ireland, manufacturers and producers provide GM potatoes at affordable prices [42], which demonstrates that the GM food industry is resilient against premium prices, and that they advocate the concept as a price efficient and nutritious food choice. Therefore, in this study, we propose the following hypothesis:

**H1.**  *Price value influences consumer attitude toward GM food consumption*.

**H2.**  *Nutritional value influences consumer attitude toward GM food consumption.*

### 2.3. Social Value

Social value is the perceived usefulness derived from social affiliation with individuals or groups apart from their status and prestige [29,35]. Social values substantially impact individual perceptions to reshape decisions for like-minded products. For instance, usually, consumers make a purchasing decision for a new product based on their experience or recommendations from a close circle people such as friends, colleagues, family members, and others who have the same demographic, socioeconomic, and cultural aspects [13,43]. These high social values transmit society acceptance and self-image enhancement to consumers [44], impacting their inclination to purchase a specific product or service [45].

However, globally, consumers have seen genetically modified organisms (GMOs) and GM food as controversial technological products, possibly due to social and digital media rumors, contentious debates on GM food [40], and the earlier scientific community’s division over the technology [46]. In short, society opposes technological developments that erode its fundamental values [47]. Therefore, “social value” as a construct significantly becomes an essential indicator of perceived social pressure (Lucht, 2015), external social pressure to achieve the adoption willingness for GM food, and motivation to establish standards based on self-evaluative rewards or penalties [48]. Moreover, in the food context, environmental and social input contributes incrementally to developing favorable or unfavorable consumer food attitudes toward the desire to eat [49]. Based on these analyses, we hypothesize that:

**H3.**  *Social value influences consumer attitudes toward GM food consumption*.

### 2.4. Emotional Value

A person’s emotional value has been described as how one feels about and experiences a particular event or circumstance and how that affects his initial natural perception and decisions [29,50,51]. From a food perspective, emotional value represents the consumption experience of the specific products [31,50], and it is basically divided into two distinct views, i.e., hedonic and utilitarian [44,51]. The hedonic view is more related to the analytical style in which an individual focuses on the practical, logical, and calculated decision [52] to adopt a specific food product, while the utilitarian view is linked with the overall satisfaction including pleasure and happiness [53] that could be experienced by consuming a specific food product. As previously stated, utilitarian and hedonic views in the emotional value construct are vital since the desire to consume is a combination of logical and subjective elements [52,54].

According to Bei et al. (1995), 89.1% of respondents purchased recyclable items to conserve and improve the environment. This emotional value of environmental safety influences consumer behavior (Finch, 2006; Lin & Huang, 2012) to prefer ecological and environmentally friendly food products. However, the previous literature has indicated that genetically modified organisms (GMOs) were harmful to the environment as compared with the traditional food varieties [55,56,57]. On the contrary, [58] explained that GM food and crops (including GM trees) were highly recommended for cultivation in droughts and harsh environmental conditions, without using pesticides, and therefore, helped to maintain and protect the ecological system and environment [13]. Therefore, environmental and ecological concerns can influence consumer attitudes [59] and consumers are much more sensitive toward these concerns [60,61], which are vital aspects to consider. Moreover, consumers’ perceptions of GMOs are a mix of positive, negative, and neutral views; consumer knowledge of GMOs has grown steadily over the years. Thus, we propose the following hypothesis:

**H4.**  *Ecological value influences consumer attitude toward GM food consumption*.

### 2.5. Epistemic Value

Epistemic value is defined as "value derived from the ability to deliver something new or distinct" [29] about a product or service that comes from the desire for knowledge it arouses in consumers due to the novelty of the product or service and the intrigue generated by its unique features [31,56]. Consumer knowledge is a vital determinant for the consumer purchase decision: consumers with better product knowledge are more analytical, conscious and educated about their adoption of new products and purchase decisions [38,62,63]. For instance, Haque, et al. [64] explained that non-muslims accept and adopt halal food after having the labelling knowledge and its benefits; Koenig-Lewis, et al. [65] elaborated that it happened because of perceived situational traits and product qualities. From the GM food perspective, governing bodies [66] and commercial organizations [67] have declared GM foods are safe and environmentally friendly, while genetically modified organisms remain a contentious concept for the general public [16,38]. Therefore knowledge becomes a pivotal trait to investigate, especially in light of the negative public opinion of genetically modified foods [68] and urges them to use their knowledge to make rational decisions based on facts linked with health concerns [69], not rumours. Thus, we hypothesize:

**H5.**  *Perceived GM Knowledge influence consumer attitude toward GM food consumption*.

### 2.6. Food Attitude and Willingness to Consume

Attitude has been described as “the evaluative influence of good or negative sentiments of individuals on their intention to engage in a certain behavior” [70] when it comes to forecasting behavioral intention and willingness to consume GM food. Notably, consumers’ actual consumption behaviors have been influenced by their attitudes regarding their willingness to consume [71] GM food. Furthermore, attitude plays a crucial role in the inclination to consume GM food [12,72]. Thus, we theorize the following:

**H6.**  *Food attitude has a significant effect on consumer willingness to consume GM food*.

**H7.**  *Food attitude significantly mediates the relationship between functional value (price) and willingness to consume GM food*.

**H8.**  *Food attitude significantly mediates the relationship between functional value (nutritional value) and willingness to consume GM food*.

**H9.**  *Food attitude significantly mediates the relationship between social value (social approval) and willingness to consume GM food*.

**H10.**  *Food attitude significantly mediates the relationship between emotional value (ecological value) and willingness to consume GM food*.

**H11.**  *Food attitude significantly mediates the relationship between epistemic value (perceived GM knowledge) and willingness to consume GM food*.

### 2.7. Ethnocentrism, Consumption Value, and Willingness to Consume GM Food

Consumer ethnocentrism relates to customers’ attitudes about prioritizing their own country’s products as compared with other countries’ products [73]. The idea of ethnocentrism includes the emotional component of purchasing imported items and the ramifications of such a decision as a risk to native businesses or even national security [74,75]. However, previous research has reported fascinating facts, such as in developed countries, people seem less ethnocentric than in emerging countries [76,77] because of cultural beliefs and socioeconomic background [78]. On the contrary, according to Li, et al. [79], ethnocentrism was less in emerging economies because the imported goods were perceived as having a superior quality or a superior social position. In the context of food, Cleveland, et al. [80] revealed that ethnocentrism and hedonistic consumption positively correlated to the consumption of the local traditional foodstuffs such as locally made ready-to-eat products. For instance, [81] studied Latino immigrants’ preferences for maize flour, a staple in their diets, and found that customers with intense ethnocentrism preferred their own country’s flour as compared with that of another country because of unfamiliarity and personal values.

Despite these facts, consumers’ purchasing attitudes also change because of many other elements such as price and quality (functional value), social approval (social value), ecological value (emotional value), and perceived knowledge (epistemic value). More specifically, consumers’ ethnocentric shifts arise based on consumer functional values in a crisis such as COVID-19 when consumers prefer highly nutritious food options [38] regardless of the premium prices [37] and their manufacturing country. However, consumers are reluctant to pay the premium prices for nutritious GM food [39] because of the lack of trust and controversies [40,41] regardless of their manufacturing country. Therefore it seems challenging for GM food producers to meet the high requirements of consumers in the entire world at affordable prices, such as producers in Ireland providing the GM potato at affordable prices [42] because they have already seen a significant reduction in other food sector sales. Likewise, social, emotional, and epistemic values significantly influence consumers’ ethnocentric behaviors, affecting their willingness to consume GM food. In summary, social approval is a strong force (Lucht, 2015), their beloved, emotional values orignate from feelings, and the epistemic value is perceived GM knowledge to self evaluate negative perception about GM food and the manufacturing country. Thus we propose the following:

**H12.**  *Consumer ethnocentrism significantly mediates with functional value (price) and willingness to consume GM food.*

**H13.**  *Consumer ethnocentrism significantly mediates with functional value (nutritional value) and willingness to consume GM food*.

**H14.**  *Consumer ethnocentrism significantly mediates with social value (social approval) and willingness to consume GM food*.

**H15.**  *Consumer ethnocentrism significantly mediates with emotional value (ecological value) and willingness to consume GM food*.

**H16.**  *Consumer ethnocentrism significantly mediates with epistemic value (perceived GM knowledge) and willingness to consume GM food*.

### 2.8. Consumer Animosity, Consumption Value, and Willingness to Consume GM Food

Consumer animosity is a type of anti-consumer behavior toward a foreign product, service, or brand that aims to change public welfare by raising awareness of a public problem and influencing organizational or governmental behaviors of the country with whom there is a conflict [82,83]. Animosity refers to hostility directed towards specific communities due to historical, economic, political, or cultural circumstances, among other things. Animosity might also result from two nations sharing a border [84]. Cui, et al. [85] found that when customer animosity was high, global companies utilized price-related marketing techniques (lower the price and give a discount) to counteract the negative effect of animosity by the consumers trade-off between animosity and price. A company from a less favorable country might be a consumer’s final choice set by counteracting the negative effect of animosity.

However, a study by [86] found that French consumers were willing to pay a high price according to their nutritional value. Other researchers have found that parents in Germany who participated in research were willing to pay a higher price to safeguard their children’s health against mycotoxins [87]. In this study, consumer animosity mediated the relationship between functional value (price and nutritional value), social approval (social value), ecological value (emotional value), perceived knowledge (epistemic value), and willingness to consume GM food. When it comes to consumer animosity and price, we argue that consumers will think twice about buying a product, especially when the price is high. However, when it comes to consumer animosity and nutritional value, consumers are willing to pay for a GM food product that has nutritional value and does not harm their health [37].

Moreover, social approval is crucial for a massive society to reach a goal easily because it plays with emotions. In the case of consumer animosity, people can quickly change their emotions due to their patriotism and social affiliation with local products, but this can also favor foreign products that provide high quality, especially in the GM food context [38]. Similarly, the COVID-19 crisis raised concern and affected the perception and feelings of the young generation to consider environmentally friendly and healthy food products [88] through utilizing their knowledge based on their nutritional values and contribution to the ecological system, such as GM food [39] providing all of these things at affordable prices [42]. Thus, we propose:

**H17.**  *Consumer animosity significantly mediates with functional value (price) and willingness to consume GM food*.

**H18.**  *Consumer animosity significantly mediates with functional value (nutritional value) and willingness to consume GM food*.

**H19.**  *Consumer animosity significantly mediates with social value (social approval) and willingness to consume GM food.*

**H20.**  *Consumer animosity significantly mediates with emotional value (ecological value) and willingness to consume GM food*.

**H21.**  *Consumer animosity significantly mediates with epistemic value (perceived GM knowledge) and willingness to consume GM food*.

## 3. Methodology

### 3.1. Data Sample and Collection

We followed the quantitative approach and employed a structured questionnaire to collect data for evaluating the proposed relationships between November 2021 and March 2022 from American and local Chinese consumers. To ensure the study’s credibility, we restricted participation to Chinese and American nationals, excluding tourists, international students, and foreign business people on tourist or temporary visas.

Secondly, we decided to collect the data from outside of the supermarkets, where it was easy to find GM food products. GM food is a unique concept; not everyone is familiar with this genetically modified food. It was essential to find the right populations that could be suitable to respond to these proposed relationships. Therefore, we visited local markets, small and large shops, restaurants, motels, and marts, but we did not find any shelves which were straightforwardly labeling GM food products. Then, we asked questions to the sales persons, managers, and shop owners about the GM food products, and surprisingly, many of them were unfamiliar with these kinds of food products. Next, we decided to move towards the supermarkets where GM food products were available and representatives of the superstores such as metro, Wal-Mart, Hyper-mart, Carrefour, Wu-Mart, Freshhema, Lianhua, Yonghui, RT-Mart, China Resources Vanguard (CR Vanguard), Amazon (Online And Physical Stores), Costco Wholesale Corporation, The Kroger Co., Albertsons Cos. Inc., Ahold Delhaize USA, and Meijer Inc., were familiar with GM food products. Additionally, supermarkets also had high-quality imported products, which allowed us to determine the participant response to consumer animosity and ethnocentrism, and explained why we chose the traditional approach for collecting data regardless of the online platforms. For this purpose, we hired participants who were experts in the food science field and who were willing to collect the data from the consumers outside of the supermarkets through face-to-face conversations (face-to-face conversations meant we only tried to give them basic information about this research project to convince them to participate in the project, which was vital to serving the community) and by providing them with questionnaire QR codes. We also provided small monetary benefits to the individuals, i.e., participants who helped to collect data from different places in China and USA. We did not pay any money to the citizens who filled out the questionnaires. Consumers either volunteered to fill out the questionnaire or they chose not to participate. There were no other means involved to convince them for data collection.

Third, in China, we faced many restrictions about travelling from one place to another because of COVID-19. We could not visit every single place by ourselves, which was very challenging and time-consuming for data collection; therefore, we offered monetary benefits to the participants who helped us to collect the data. Finally, we presented the questionnaire in English in the USA and in Chinese in China using the back-translation approach to ensure semantic equivalence between the English and Chinese versions of the questionnaire [1,89]. Then, we distributed translated questionnaires with QR codes on the spot to Chinese consumers in China and American consumers in USA to collect the actual data to achieve our research objectives.

Initially, we carried out pilot research and gathered data from 400 different consumers from USA and 300 consumers from China. After collecting feedback from the hired participants, we made a few modifications to the questionnaire based on their expertise in this field (see detailed questionnaire in the Appendix A). With the assistance of the hired participants, we delivered 1600 questionnaires in both the United States and China outside of supermarkets after making the necessary adjustments. We obtained 1340 valid responses out of 1600 from the USA; in the initial 400 collected questionnaires, prior to adjustments, 200 incomplete responses, and 60 outlier responses. We obtained 1065 valid responses out of 1600 from China; the 300 pilot responses provided 180 incomplete responses and 25 outliers responses. The response rate for the USA was 83.75%, and for China, it was 66.56%.

We utilized all the constructs from the previous studies, such as the functional value of nine items (4 for price value and 5 for nutritional value), the social value of four items (social approval), the emotional value of three items (ecological values), the epistemic value of four items (perceived GM knowledge), food attitude of four items, and willingness to consume GM food of five items adapted [31,35,84]. Furthermore, because there was no war between China and the United States, the mediation variable, consumer enmity, was divided into three animosity dimensions and ten items: economic animosity, public animosity, and government animosity that were adopted from [90] and five consumer ethnocentrism items from [91]. A 5-point Likert scale was used for the responses to all construct items, from 1 strongly agree to 5 strongly disagree. Table 1 presents a detailed measurement scale.

### 3.2. Data Analysis

In this research, we employed a quantitative approach to analyze the proposed relationships. For this reason, we utilized structural equation modeling (SEM), the most potent statistical multivariate technique, to determine the relationships between observed and latent variables along with continuously recorded measurement errors [13,40]. SEM is a combination of a measurement model and a structural model. At the same time, a measurement model includes a factor analysis, confirmatory factor analysis, model fitness indices, and common method bias [40,92]. A factor analysis assists researchers to reduce a large number of cointegrated variables into a fewer variables, while a confirmatory factor analysis ensures convergent and discriminant validity and the issue of multicollinearity [92,93]. Similarly, model fitness indices and common method biases help us to measure whether a proposed model is acceptable for regression analysis [92,93].

Moreover, structured models are used in research to determine the strength of connections between latent variables (with respect to path coefficients). Wright (1920, p. 329) proposed that path coefficients could be used to quantify the relative significance of many paths of causation to explain an outcome [94]. Each coefficient in the structural equation was calculated while the other potential sources of variation were considered. Therefore, unlike conventional multiple regression models, all coefficients for dependent variables were computed concurrently.

## 4. Results

### 4.1. Measurement Model

#### 4.1.1. Convergent and Discriminant Validity

After ensuring the construct validity, we examined the convergent and discriminant validity in both cases, i.e., USA and China, via reliability, validity through confirmatory factor analysis (CFA) [95], and the Heterotrait-Monotrait (HTMT) ratio of correction technique [96]. For this purpose, we used AMOS 25 and SMART PLS 3 to analyze the measurement model along with all the construct items. In both methods, the results were satisfactory and acceptable. Appendix A shows that the factor loadings for USA and China are above the threshold of 0.7 remaining are removed to ensure the true nature of the proposed model. Moreover, Table 1 represents the average variance obtained (AVE) and composite reliability (CR) values, which show that, for all constructs, the CR values are greater than 0.792 and the AVE values are above the 0.5 thresholds [95,97], confirming that there is no convergent validity issue. Similarly, the maximum shared variance (MSV) values are lower than the AVE values, highlighting no discriminative validity [98,99].

Furthermore, we also performed another test Heterotrait-Monotrait (HTMT) ratio of correction technique [96] to determine if there were any discriminant validity concerns. Table 2 shows that construct values do not exceed the HTMT [100] cut-off values and also indicate no multicollinearity issue.

#### 4.1.2. Evaluation of Model Fitness Indices

The SmartPLS 3 software only assessed the standardized root mean square residual (SRMR), which was not enough to show the model’s fitness; therefore, we relied upon the AMOS 25 to check the overall model fitness indices such as the comparative fit index (CFI), the root mean square error of approximation (RMSEA), and model chi-square and degree of freedom (CMIN/DF) for statistically model acceptability to perform the path analysis test. Table 3 represents the model fitness outcome which satisfies the cut-off criteria [101].

#### 4.1.3. Common Method Bias

Common method bias can be a major problem that is overlooked by a researcher that can cause false results and implications on the entire outcomes of a proposed model. Therefore, it was necessary to investigate this concern by using the Harman’s one-factor test [102] that explained the single factor variance, which should be less than 50% to ensure that there were no common method biases [103]. In this study, the single factor variance was 18.871% for the USA and 15.123% for China, which was less than the cut-off value [102]. Moreover, we also examined the common method biases through the variance inflation factor (VIF) that represents the multicollinearity problems among the proposed variables. In our study, the VIF range for the USA was 1.241–2.369 and, for China, it was 1.309–2.686, which was less than the cut-off value of three [104], and showed that there were no common method biases and multicollinearity issues [105]. Furthermore, we followed the Ghufran et al. (2022) approach to identify any substantially high correlations among the constructs; the results showed no high correlation between constructs (see Table 1 and Table 2). Finally, we can conclude that our study has no common method biases.

### 4.2. Structural Model

We used the Smart PLS 3 for SEM (structural equation modeling) to analyze the structural and measurement model because it was well-suited for complicated models, structures and formative constructs [106,107]. The structural model explained the proposed connection between the constructs. Table 4 represents the coefficient of predictors with dependent variables by using the 5000 bootstrap sampling technique.

In Table 4, the results indicate that consumption values, including functional value price (H1) and quality (H2), social value (H3), and emotional value (H4), positively influence consumer willingness to consume GM food (β = 0.178, 0.188, 0.316, 0.239, *p* < 0.001), except epistemic value (H5) that is non-significant in the case of USA (β = 0.045), while all consumption values positively affect consumer willingness to consume GM food in the Chinese context (β = 0.073, *p* < 0.05, 0.122, 0.230, 0.093, 0.077, *p* < 0.001). Similarly, as mediators of food attitude, consumer animosity and consumer ethnocentrism positively effect consumer willingness to consume GM food in the context of the USA (β = 0.269, *p* < 0.001, 0.051, *p* < 0.05, 0.091, *p* < 0.01), while consumer animosity is non-significant (β = 0.111) in the Chinese context and the others are positively significant (β = 0.253, 0.135, *p* < 0.001).

Furthermore, food attitude as a mediator positively partially mediates the consumption values, including functional value price (H7) and quality (H8), social value (H9), emotional value (H10), and epistemic value (H11) between consumer willingness to consume GM food in both cases: Chinese (β = 0.053, 0.036, 0.041, 0.079, 0.043, *p* < 0.001) and USA (β = 0.056,0.032, 0.044, 0.096, *p* < 0.001), except epistemic value (H11) which is fully mediating by food attitude (β = 0.040, *p* < 0.001). Similarly, consumer ethnocentrism, as a mediator, does not mediate the relationship between consumption values and consumer willingness to consume GM food and all hypothesis from Hypotheses 12 to 16 are rejected in the case of the USA (β = 0.021, 0.022, 0.026, 0.006, 0.014), while, in the case of China, all are accepted (β = 0.028, 0.035, *p* < 0.05, 0.035, *p* < 0.001, 0.018, 0.014, *p* < 0.05) and present partial mediation between consumption values and consumer willingness to consume the GM food. Finally, consumer animosity partially mediates the relationship between consumption values and consumer willingness to consume GM food in the case of the USA, while there is no mediation in the case of China.

Additionally, Table 4 provides the R2 square values for each sample, which demonstrates the variance explained by the proposed model. The R^2^ square values of consumer animosity are 0.630 and 0.678, consumer ethnocentrism 0.790 and 0.803, food attitude 0.830 and 0.843, and willingness to consume GM food 0.810 and 0.837, for USA and China, which are over the 40% threshold for model appropriateness, indicating excellent capability for investigating the influence between consumption values and food attitude, consumer animosity, consumer ethnocentrism, and consumer willingness to consume GM food in a crisis such as COVID-19. Moreover, as per the blindfolded result, Q2 is more than the cut-off value of zero in both cases of the USA and China (see Table 4); it can be concluded that each model possesses an appropriate predictive quality in accordance with the findings of previous studies [1,108].

## 5. Discussion and Conclusions

In this study, we examined the socioeconomic and psychological aspects of COVID-19 that have influenced consumer attitudes, behaviors, and willingness to buy genetically modified (GM) foods in light of the theory of consumption values. In the case of the United States and China, it is interesting to study attitudes about GM food products from the perspective of consumer animosity and ethnocentrism in both economic powers because the United States dominated the GM food industry from 1996 to 2017 by producing nine GM crops on 75 million hectares (soybeans, maize, cotton, alfalfa, canola, sugar beets, potato, apples, squash, and papaya) [6] and by exporting to China [7]. At the same time, China is a prominent importer of GM food products. More precisely, China imported 27 million tons of GM corn and 100 million tons of soybeans [109] in 2021 [110]. Our results show that Chinese consumers do not have animosity toward GM food products. Thus, Hypotheses 17–21 are rejected in the case of China; hence, consumer animosity does not mediate the relationship between consumption values and consumers’ willingness to consume GM food. There is a very obvious reason, i.e., the Chinese government cannot afford any negative sentiment in the public about GM food products when they are heavily dependent on the USA and other GM food producing countries such as Brazil, Argentina, and Ukraine to meet consumer food demand [110].

Similarly, back in 2004, when China went from being a net exporter of food to a net importer [111], and since then, the difference between imports and exports has grown [112]. The Chinese government is deeply concerned about the threat to agriculture production, given the country’s vast population and its history of food scarcity, including significant crop failures [112]. For this reason, the Chinese government believes that expanding the agriculture sector is an integral part of accomplishing its food security targets, as food insecurity is a source of political turmoil and there is a perception that food can be used as a strategic instrument by other countries to intimidate national security in China [112,113,114]. Failure to boost agriculture production will increase China’s global imports [112]. Similarly, before the COVID-19 crisis, the world was already facing a food shortage problem; according to a 2019 report by the United Nations, 690 million people were malnourished, representing 8.9 per cent of the global population [2], and the COVID-19 outbreak doubled that number, placing an additional 130 million people at risk of serious famine by the end of 2020 [3]. As forecasted by the World Food Program (UN), the world’s population is expected to exceed 9 billion by 2050, making GM food a viable answer for reducing hunger in developing and underdeveloped countries [3].

From the perspective of the USA, consumer animosity partially mediates the relationship between consumption values and consumer willingness to consume GM food; therefore, Hypotheses 17–21 are accepted in the USA context. However, in this situation, the organizations utilized different strategies to minimize the adverse effect of animosity. For example, Cui, Wajda and Hu [85] found that organizations usually used price-related marketing methods (reduce the price and give a discount) and offered high nutritional value [86] to counteract the negative influence of animosity, owing to consumers’ animosity price and quality trade-off. During COVID-19, when it comes to consumer animosity and nutritional value, consumers have been prepared to pay for GM food products and organic food [1] as long as they had nutrititional value and did not negatively impact their health [37] and environment [88].

We also have exciting results regarding consumer ethnocentrism in the case of the USA, which represent no mediation between consumption values and consumer willingness to consume GM food. Therefore, Hypotheses 12–16 are rejected. Our study’s findings were aligned with previous studies that have presented that developed nations’ consumers were less ethnocentric than developing nations [76,77] due to their social, cultural, and economic beliefs and background [78,115]. However, negative sentiments can be triggered and utilized by economies for their political benefits, such as in the Trump administration’s cold war between the USA and China when the U.S. president banned many Chinese companies such as Huawei and software applications such as TikTok [116,117]. However, this political tug of war did not last forever, and the situation became normal after a while. Thus, these implications can not be applied to food items which are considered to be necessities [118]; at the same time, the Chinese administration renewed the agreement and approved imports of ten GM food products, including A5547-127 soybean; MON89788 soybean; H7-1 beet; Oxy-235 canola; T25 corn; T45 canola; 305,423 soybeans; Ms8Rf3 canola; and 305,423 × GTS40-3-2 soybean [109], with a U.S. trade deal.

In the case of China, contrary to the USA, consumer ethnocentrism partially mediates the consumption values, and consumers’ willingness to consume GM food; therefore Hypotheses 12–16 are accepted. Notably, Li, Yang, Wang and Lei [79] found that consumer ethnocentrism seemed minor in developing countries such as China, where consumers believed that imported goods had superior quality or a higher social position. Specifically, consumer ethnocentrism changes under consumption values, including consumer functional values during a crisis such as COVID-19, when consumers prefer healthy food value options [38] despite the high prices [37] and their manufacturing country [37]. However, this situation is different in the case of GM food, where consumers are unlikely to pay premium prices for healthy GM food [39] due to a lack of confidence and controversies [40], regardless of the manufacturing country. Therefore, it appears to be difficult for GM food producers to meet the high demand of consumers worldwide at reasonable prices, such as the manufacturers in Ireland who provide GM potatoes at reasonable prices [42], given that organic food sales in Ireland have declined significantly [119]. Similarly, social value, emotional value, and epistemic value have substantial impacts on the ethnocentric behaviors of consumers and their readiness to ingest GM food. While social approbation is a powerful force (Lucht, 2015) from their loved ones, emotional value is feeling, and epistemic value is perceived GM information for self-evaluation of the unfavorable view of GM food and the manufacturing country.

### 5.1. Practical Implications

#### 5.1.1. For Decision-Makers

Globally, as of 2021, the Chinese population was estimated by the U.S. Census Bureau to be 1.4 billion, with USD 151. billion goods exported to USA, while USD 506.4 billion are imported from USA, and maximum imports linked to food markets [120]. Similarly, before the COVID-19 crisis, the world was already facing a food shortage problem. According to a 2019 report by the United Nations, 690 million people were malnourished, representing 8.9 per cent of the global population [2,3]; the COVID-19 outbreak doubled that number, placing an additional 130 million people at risk of severe famine by the end of 2020 [2,3]. As the World Food Program (UN) forecasted, the world’s population is expected to exceed 9 billion by 2050, making GM food a viable answer for reducing hunger in developing and underdeveloped countries [2,3]. These figures indicate that China and other developing nations still have a long way ahead to fill the food import and export gaps and its shortage problem. It is a golden opportunity for decision-makers and industry leaders to jump into this potential food segment, which could help governments to overcome such problems and even augment profit maximization endeavours through the high yield of GM crops. Even though the COVID-19 crisis has substantially impacted consumer perceptions and attitudes about food products, consumers are more inclined toward healthy and safe food and are ready to pay high prices [1]. Similarly, the World Health Organization has reported that approved GM crops and food products are safe, healthy, and full of nutrients.

#### 5.1.2. For Managers

Previous research has shown that consumers were concerned about GM food worldwide, especially in China and the USA [1,40]. Similarly, Cui and Shoemaker [14] reported that only 11.9% of Chinese consumers had positive perceptions of GM food, while in the USA, 51% of consumers believed that GM food was healthy and nutritious [15]. Regardless of these facts, the Chinese government is importing GM food products such as GM maize, soybeans, canola, GM papaya, and rice to meet local food demands [12,13]. Additionally, COVID-19 has exposed the entire world’s food system to augmented scrutiny, and people have become more conscious regarding their health and food safety, which has compelled them to find other safe food alternatives, such as organic and GM foods relative to traditional and wild food options. There is no evidence that COVID-19 spread from the food sector [1]. Therefore, it is a window of opportunity for managers and stakeholders to gain consumer trust and to establish sustainable GM food positioning in the market of 1.4 billion people in China and, in a similar manner, to attain an increased percentage of consumers in the USA by enhancing awareness about the true nutritional value of GM food products. National recognition exists for GM food, as both countries support the idea of GM products and provide all possible support through flexible respective jurisdictions to stakeholders to invest and engage in the GM food sector [16].

#### 5.1.3. For Policymakers

This study shows that the USA is the leading producer and manufacturer of GM food, and that the U.S. agriculture sector is much stronger than the entire world. Policymakers must modify their existing policies regarding GM food product imports and engage with foreign institutes to benefit from their expertise and technology along with GM food products, which will help local farmers and producers to obtain the new technology and expertise to cultivate and produce GM food products. This strategy would also help to reduce imports and dependencies from other countries.

### 5.2. Limitations and Future Directions

This study has certain limitations and future directions. First, this study is comparative between the USA and China, and scholars should consider other countries or regions such as the European Union (EU) and African Union (AU) to investigate this exciting model from a diverse angle. Second, scholars should consider some essential constructs to examine the impact of consumption values on consumer willingness to consume GM food in the presence of cultural aspects, consumer buying power, political system, and personal traits. Third, we employed convenient sampling techniques as compared with representative sampling techniques, and researchers should consider representative sampling techniques for future research work to investigate the proposed model.

## Figures and Tables

**Table 1:** Convergent and discriminant validity.

USA													
Constructs	CR	AVE	MSV	MaxR(H)	1	2	3	4	5	6	7	8	9
1. Consumer animosity	0.866	0.685	0.515	0.883	**0.827**								
2. Epistemic value	0.887	0.612	0.517	0.890	0.717 ***	**0.782**							
3. Emotional value	0.841	0.639	0.517	0.855	0.660 ***	0.719 *	**0.799**						
4. Consumer ethnocentrism	0.855	0.665	0.496	0.884	0.635 *	0.691 **	0.704 *	**0.816**					
5. Food attitude	0.846	0.648	0.458	0.863	0.641 **	0.677 **	0.619 **	0.665 **	**0.805**				
6. Functional value-price	0.853	0.660	0.512	0.864	0.623 **	0.715 *	0.711 **	0.627 **	0.622 **	**0.812**			
7. Functional value-quality	0.792	0.560	0.553	0.798	0.604 *	0.708 **	0.655 **	0.637 **	0.540 **	0.638 **	**0.748**		
8. Social value	0.825	0.703	0.553	0.828	0.562 *	0.640 **	0.615 *	0.566 **	0.566 **	0.582 **	0.743 **	**0.838**	
9. Willingness to consume GM food	0.793	0.561	0.510	0.800	0.658 *	0.692 *	0.675 **	0.590 **	0.673 **	0.714 **	0.646 **	0.688 **	**0.749**
**China**													
**Constructs**	**CR**	**AVE**	**MSV**	**MaxR(H)**	**1**	**2**	**3**	**4**	**5**	**6**	**7**	**8**	**9**
1. Consumer animosity	0.795	0.506	0.041	0.861	**0.711**								
2. Epistemic value	0.917	0.649	0.11	0.918	0.186 ***	**0.806**							
3. Emotional value	0.817	0.602	0.519	0.85	0.077 *	0.252 ***	**0.776**						
4. Consumer ethnocentrism	0.813	0.598	0.519	0.864	0.041	0.277 ***	0.720 ***	**0.774**					
5. Food attitude	0.869	0.624	0.032	0.878	0.158 ***	−0.012	0.017	0.037	**0.79**				
6. Functional value-price	0.844	0.576	0.138	0.857	0.202 ***	0.332 ***	0.296 ***	0.372 ***	0.035	**0.759**			
7. Functional value-quality	0.861	0.674	0.005	0.87	−0.003	−0.009	−0.009	−0.068 †	0.022	0.048	**0.821**		
8. Social value	0.839	0.647	0.012	0.913	0.079 *	0.084 *	0.048	0.053	0.110 **	0.089 *	0.015	**0.804**	
9. Willingness to consume GM food	0.792	0.56	0.032	0.803	0.018	−0.008	−0.025	−0.083 *	0.178 ***	−0.017	−0.048	0.109 **	**0.749**

Notes: CR, composite reliability; AVE, average variance extracted; MSV, maximum shared variance; MaxR(H), maximum reliability; (H) and †, square root of AVE. † *p* < 0.100, * *p* < 0.050, ** *p* < 0.010, *** *p* < 0.001.

**Table 2:** Discriminant validity using HTMT.

USA									
Constructs	1	2	3	4	5	6	7	8	9
1. Consumer animosity									
2. Epistemic value	0.754								
3. Emotional value	0.698	0.738							
4. Consumer ethnocentrism	0.699	0.736	0.774						
5. Food attitude	0.67	0.705	0.657	0.721					
6. Functional value-price	0.693	0.733	0.76	0.709	0.67				
7. Functional value-quality	0.639	0.729	0.662	0.682	0.576	0.665			
8. Social value	0.591	0.638	0.628	0.603	0.58	0.61	0.756		
9. Willingness to consume GM food	0.684	0.702	0.691	0.651	0.704	0.743	0.652	0.684	
**China**									
**Constructs**	**1**	**2**	**3**	**4**	**5**	**6**	**7**	**8**	**9**
1. Consumer animosity									
2. Epistemic value	0.193								
3. Emotional value	0.078	0.254							
4. Consumer ethnocentrism	0.055	0.304	0.846						
5. Food attitude	0.129	0.019	0.007	0.054					
6. Functional value-price	0.24	0.341	0.329	0.391	0.026				
7. Functional value-quality	0.016	0.017	0.03	0.051	0.003	0.048			
8. Social value	0.033	0.073	0.026	0.046	0.113	0.078	0.024		
9. Willingness to consume GM food	0.022	0.02	0.049	0.073	0.184	0.006	0.06	0.117	

Note: Heterotrait-Monotrait (HTMT) ratio of correction technique.

**Table 3:** Model fitness analysis.

Data	CMIN/DF	CFI	SRMR	RMSEA
China	2.944	0.916	0.055	0.070
USA	2.099	0.92	0.05	0.057
Criteria	>1	>0.95	<0.08	<0.08

**Table 4:** Regression table.

		USA			China		
	Constructs	β	Results	Mediation Outcome	β	Results	Mediation Outcome
	**Direct relationship**						
H1	Functional value-price (FVP) -> willingness to consume GM food (WCGM)	0.178 ***	Accepted		0.073 **	Accepted	
H2	Functional value-quality (FVQ) -> WCGM	0.188 ***	Accepted		0.122 ***	Accepted	
H3	Social value (SV) -> WCGM	0.316 ***	Accepted		0.230 ***	Accepted	
H4	Emotional value (EV)-> WCGM	0.239 ***	Accepted		0.093 ***	Accepted	
H5	Epistemic value (EP) -> WCGM	0.045	Rejected		0.077 ***	Accepted	
	**Mediators**						
H6	Food attitude (FDA) -> WCGM	0.269 ***	Accepted		0.253 ***	Accepted	
	Consumer animosity (CA) -> WCGM	0.051 **	Accepted		0.111	Rejected	
	Consumer ethnocentrism (Etho) -> WCGM	0.091 *	Accepted		0.135 ***	Accepted	
	**Mediation relationship**						
	Functional value-price (FVP) -> FDA	0.208 ***	Accepted		0.206 ***	Accepted	
	FVP -> CA	0.149 ***	Accepted		0.171 ***	Accepted	
	FVP -> Etho	0.225 ***	Accepted		0.209 ***	Accepted	
	Functional value-quality (FVQ) -> FDA	0.120 ***	Accepted		0.141 ***	Accepted	
	FVQ -> CA	0.252 ***	Accepted		0.269 ***	Accepted	
	FVQ -> Etho	0.236 ***	Accepted		0.262 ***	Accepted	
	Social value -> FDA	0.165 ***	Accepted		0.163 ***	Accepted	
	SV -> CA	0.172 ***	Accepted		0.150 ***	Accepted	
	SV -> Etho	0.285 ***	Accepted		0.262 ***	Accepted	
	Emotional value -> FDA	0.357 ***	Accepted		0.313 ***	Accepted	
	EV -> CA	0.116 ***	Accepted		0.107 ***	Accepted	
	EV -> Etho	0.153 ***	Accepted		0.132 ***	Accepted	
	Epistemic value (EP) -> FDA	0.149 ***	Accepted		0.169 ***	Accepted	
	EP -> CA	0.187 ***	Accepted		0.195 ***	Accepted	
	EP -> Etho	0.068 ***	Accepted		0.102 **	Accepted	
	**Specific indirect relationship**						
H7	FVP -> FDA -> WCGM	0.056 ***	Accepted	Partial mediation	0.053 ***	Accepted	Partial mediation
H8	FVQ -> FDA -> WCGM	0.032 ***	Accepted	Partial mediation	0.036 ***	Accepted	Partial mediation
H9	SV -> FDA -> WCGM	0.044 ***	Accepted	Partial mediation	0.041 ***	Accepted	Partial mediation
H10	EP -> FDA -> WCGM	0.040 ***	Accepted	Fully mediation	0.079 ***	Accepted	Partial mediation
H11	EV -> FDA -> WCGM	0.096 ***	Accepted	Partial mediation	0.043 ***	Accepted	Partial mediation
H12	FVP -> Etho -> WCGM	0.021	Rejected	No mediation	0.028 **	Accepted	Partial mediation
H13	FVQ -> Etho -> WCGM	0.022	Rejected	No mediation	0.035 **	Accepted	Partial mediation
H14	SV -> Etho -> WCGM	0.026	Rejected	No mediation	0.035 ***	Accepted	Partial mediation
H15	EP -> Etho -> WCGM	0.006	Rejected	No mediation	0.018 **	Accepted	Partial mediation
H16	EV -> Etho -> WCGM	0.014	Rejected	No mediation	0.014 **	Accepted	Partial mediation
H17	FVP -> CA -> WCGM	0.008 *	Accepted	Partial mediation	0.019	Rejected	No mediation
H18	FVQ -> CA -> WCGM	0.013 *	Accepted	Partial mediation	0.023	Rejected	No mediation
H19	SV -> CA -> WCGM	0.009 *	Accepted	Partial mediation	0.016	Rejected	No mediation
H20	EP -> CA -> WCGM	0.010 *	Accepted	Partial mediation	0.011	Rejected	No mediation
H21	EV -> CA -> WCGM	0.006 *	Accepted	Partial mediation	0.022	Rejected	No mediation
	R^2^ Consumer animosity	0.630			0.678		
	Adjusted R^2^ Consumer animosity	0.629			0.677		
	R^2^ Consumer ethnocentrism	0.790			0.803		
	Adjusted R^2^ Consumer ethnocentrism	0.785			0.802		
	R^2^ Food attitude	0.830			0.843		
	Adjusted R^2^ Food attitude	0.828			0.843		
	R^2^ willingness to consume GM food	0.810			0.837		
	Adjusted R^2^ willingness to consume GM food	0.809			0.836		
	Q^2^ Consumer animosity	0.372			0.417		
	Q^2^ Consumer ethnocentrism	0.513			0.543		
	Q^2^ Food attitude	0.518			0.552		
	Q^2^ willingness to consume GM food	0.503			0.550		
	VIF range	1.241–2.369			1.309–2.686		

Note: * *p* < 0.050, ** *p* < 0.010, *** *p* < 0.001.

## Data Availability

Data is contained within the article.

## Appendix A. Factor Loading and Items

		**China**	**USA**
**Constructs**	**Items**	**Loadings**	**Loadings**
Functional value-quality	Overall, I think GM food provides a variety of ingredients.	0.882	0.866
	Overall, I think GM food provides good quality ingredients.	0.790	0.778
	Overall, I think GM food provides appealing flavors.	Removed (0.543)	0.816
	Overall, I think GM food is tasty.	Removed (0.490)	0.810
	Overall, I think GM food provides a high standard of quality.	0.837	0.690
Functional value-price	GM food is reasonably priced.	0.88	0.872
	GM food offers value for money.	0.757	0.73
	GM food is a good product for the price.	0.877	0.875
	GM food is economical	Removed (0.321)	0.721
Social value	Buying GM food helps me feel acceptable	0.845	0.818
	Buying the GM food improves the way that I am perceived	0.828	0.811
	Buying the GM food makes a good impression on other people	0.824	0.791
	Buying the GM food gives its owner social approval	0.819	0.802
Emotional value	Buying GM food instead of conventional food; feels like making a good personal contribution to something better	0.847	0.845
	Buying GM food instead of conventional food feels morally right	0.807	0.782
	Buying GM food instead of conventional food makes me feel better	0.849	0.832
Epistemic value	Before buying the GM food, I obtain substantial information about the different manufacturers and producers of GM food	0.821	0.793
	I would acquire a great deal of information about the different manufacturers and producers before buying GM food	0.788	0.778
	I am willing to seek out novel information about GM food products	0.804	0.792
	I like to search for the new and different GM food products	0.837	0.815
Food attitude	Overall, I think GM food is hygienic	0.818	0.82
	Overall, I think GM food makes me healthy	0.807	0.786
	Overall, I think GM food is safe	0.814	0.802
	Overall, I think GM food provides good nutrition	0.827	0.826
Willingness to consume GM food	I make a special effort to buy GM food products (that are environment friendly) as compared to the conventional food	0.816	0.804
	I have switched to conventional food products for ecological reasons	0.797	0.765
	When I have a choice between two equal food products, I purchase the one that is less harmful to other people and the environment	0.811	0.789
	I make a special effort to buy products using fewer chemicals such as pesticides and are environmentally friendly	0.817	0.800
	I have avoided buying a product because it had potentially harmful environmental and health effects	0.821	0.805
Economic animosity	USA/China wants to gain economic power over China/USA	0.822	0.793
	USA/China is taking advantage of China/USA	0.701	Removed (0.53)
	USA/China has too much economic influence on China/USA.	0.690	0.730
People animosity	I am not too fond of the mentality of the Americans/Chinese	0.818	Removed (0.44)
	I feel that the people in the USA/China are hostile and not open to foreigners	0.791	0.776
	My experiences with Americans/Chinese are negative	Removed (0.324)	0.823
Politics/government animosity	I am not too fond of this US/Chinese government policy	0.794	0.771
	I am not too fond of the political system in the USA/China	0.750	0.719
	There is too much corruption in the USA/China	Removed (0.118)	0.716
Consumer ethnocentrism	Chinese/American products, first, last, and foremost	0.828	0.813
	Purchasing foreign-made products are un-Chinese/American	Removed (0.211)	0.812
	It is not right to purchase foreign products because it puts the Chinese/American out of jobs	0.815	0.789
	We should purchase products manufactured in China/USA instead of letting other countries get rich from us	0.817	0.830
	We should buy from foreign countries only those products that we cannot obtain within our own country	0.839	0.731
